# Antimicrobial Resistance in the Food Chain: Bridging Knowledge Gaps for Effective Detection and Control

**DOI:** 10.3390/antibiotics15030262

**Published:** 2026-03-03

**Authors:** Emílio Gomes, Tomás Gonçalves Mesquita, Patrícia Serra, Daniela Araújo, Carina Almeida, António Machado, Ricardo Oliveira, Joana Castro

**Affiliations:** 1 INIAV—National Institute for Agrarian and Veterinary Research, Rua dos Lagidos, Lugar da Madalena, Vairão, 4485-655 Vila do Conde, Portugal; up202008704@edu.fe.up.pt (E.G.); tgmesquita@ucp.pt (T.G.M.); id12338@uminho.pt (P.S.); daniela.araujo@iniav.pt (D.A.); carina.almeida@iniav.pt (C.A.); ricardo.oliveira@iniav.pt (R.O.); 2 LEPABE, AliCE, Faculty of Engineering, University of Porto, Rua Dr. Roberto Frias, 4200-465 Porto, Portugal; 3 CEB—Centre of Biological Engineering, University of Minho, Campus de Gualtar, 4710-057 Braga, Portugal; 4 Laboratorio de Bacteriología, Instituto de Microbiología, Colegio de Ciencias Biológicas y Ambientales COCIBA, Universidad San Francisco de Quito USFQ, Calle Diego de Robles y Pampite, Quito 170901, Ecuador; amachado@usfq.edu.ec; 5 Department of Biology, Faculty of Sciences and Technology, University of the Azores, Rua da Mãe de Deus, 9500-321 Ponta Delgada, Portugal; 6 CIBIO, Research Centre in Biodiversity and Genetic Resources, InBio Associate Laboratory, BIOPOLIS Program in Genomics, Biodiversity and Land Planning, University of the Azores, Rua da Mãe de Deus, 9500-321 Ponta Delgada, Portugal

**Keywords:** antimicrobial resistance, food chain, food safety, One Health, whole genome sequencing, AMR mitigation strategies

## Abstract

Antimicrobial resistance (AMR) poses a critical global public health threat, with the food chain serving as a significant transmission route connecting animals, environment, and humans. This review adopts a One Health perspective to analyze the key drivers of AMR dissemination across animal agriculture, aquaculture and food processing. We evaluate detection methodologies, contrasting the regulatory gold standard of culture-based phenotypic testing with rapid molecular advancements, including Whole Genome Sequencing (WGS), metagenomics, and emerging CRISPR-Cas diagnostics. While molecular tools offer unprecedented speed and resolution, challenges such as matrix interference, the viable but non-culturable (VBNC) state, and the genotype-phenotype disconnect remain. Finally, integrated mitigation strategies are also described, ranging from on-farm antimicrobial stewardship and innovative biofilm control to consumer hygiene practices. It is essential to bridge the technical and regulatory gaps in AMR surveillance in order to develop effective interventions and ensure a safer food system.

## 1. Introduction

Antimicrobial resistance (AMR) is among the top ten global public-health threats, with >1.27 million deaths attributed to resistant bacterial infections in 2019 [1]. Regionally, this burden is disproportionately higher in low- and middle-income countries (LMICs), where fragile sanitation infrastructures and less regulated access to antimicrobials exacerbate transmission risks [1]. Within the food chain specifically, the global consumption of antimicrobials in food-producing animals is projected to rise dramatically, driven largely by shifting dietary patterns and intensified farming practices in developing regions [2]. This agricultural use significantly amplifies the regional and global spread of foodborne resistant pathogens, translating into severe economic burdens, higher healthcare costs, and increased mortality across interconnected ecosystems [2,3]. The growing prevalence of antimicrobial-resistant microorganisms reduces the effectiveness of current treatments, resulting in prolonged illnesses, higher healthcare costs, and increased mortality [3]. Beyond clinical settings, AMR poses a significant challenge to food safety and security, as resistant bacteria and genetic determinants can move seamlessly across interconnected ecosystems, from farms and food production systems to the wider environment and ultimately reaching consumers [4]. The food chain, spanning primary production, processing, distribution, and consumer handling, constitutes a major, yet largely under-quantified, exposure route for AMR emergence, amplification, and dissemination [5].

At farm level, the use and misuse of antimicrobials in livestock [2], aquaculture [6], and crop production [7] create selective pressure that promotes the development of resistant bacteria [8]. These organisms and their genetic determinants can persist in manure, soil, and water, facilitating environmental dissemination and horizontal gene transfer among microbial communities. During food processing and distribution, cross-contamination events and inadequate hygiene practices can further spread resistant strains, and consumers may be exposed through contaminated food products or contact surfaces, thereby perpetuating the transmission cycle [8].

Despite growing awareness, substantial gaps remain in the detection and control of AMR within the food chain. Conventional culture-based susceptibility testing remains the gold standard for AMR monitoring in foods, but time-to-result (48–72 h) and limited throughput hinder proactive risk management [9]. Furthermore, conventional monitoring approaches often fail to capture the complexity of resistance dynamics, particularly those associated with microbial communities embedded in biofilms [10]. Biofilm-associated AMR represents a critically under-recognized challenge, as biofilms provide a protective environment that enhances bacterial persistence, resistance, and tolerance to antimicrobials and disinfectants in food industry [11]. Modern molecular assays, such as qPCR, digital-PCR, and shotgun metagenomics, substantially reduce detection times (<8 h) and allow multiplexed quantification of diverse genetic determinants in a single run [12]. Emerging on-site technologies, such as CRISPR-Cas12 fluorescence (DETECTR) [13], nanopore sequencing, and AI-supported biosensors, now enable near-real-time generation of actionable data from farm, processing, or abattoir swabs [14].

Rather than proposing conclusive operational solutions, this paper aims to provide an updated synthesis of available evidence regarding AMR detection and control within the food chain. By organizing and critically analyzing current methodologies, we seek to identify key trends, highlight specific technological patterns, and interpret existing gaps to support evidence-based decision-making and strategy development.

## 2. Methodology

**Figure 1:**
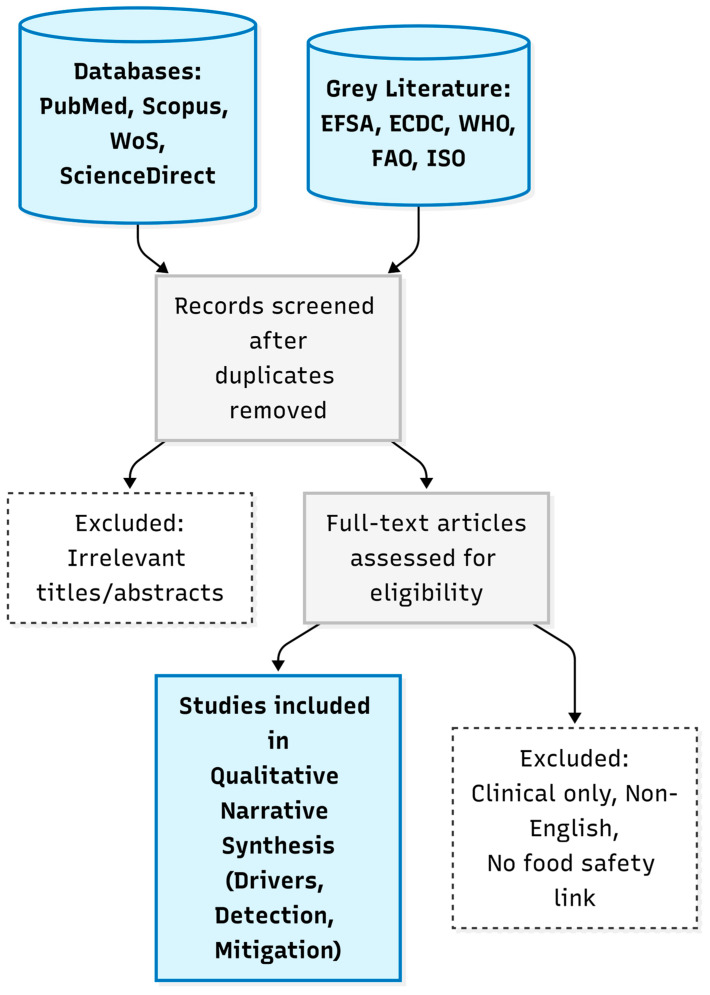
PRISMA flow diagram illustrating the literature search and selection process. The diagram details the identification of records from scientific databases (PubMed, Scopus, Web of Science, ScienceDirect) and grey literature (e.g., EFSA, ECDC, WHO), followed by the screening and eligibility phases that led to the final selection of studies for the qualitative narrative synthesis.

This paper presents a comprehensive narrative review designed to analyze the current landscape of AMR within the food chain, with a specific focus on bridging the gap between detection capabilities and mitigation strategies from a One Health perspective. Although structured as a narrative synthesis, the literature search and study selection processes were guided by the Preferred Reporting Items for Systematic Reviews and Meta-Analyses (PRISMA) framework to ensure methodological transparency, rigor, and reproducibility. To ensure a rigorous synthesis of available evidence, a structured literature search was executed between November 2025 and February 2026. Primary data were identified across major scientific databases, including PubMed (NCBI), Scopus, Web of Science, and ScienceDirect as shown in Figure 1. These sources were supplemented by grey literature, technical guidelines, and surveillance reports from official regulatory bodies, specifically the European Food Safety Authority (EFSA), the European Centre for Disease Prevention and Control (ECDC), the World Health Organization (WHO), the Food and Agriculture Organization (FAO), and the International Organization for Standardization (ISO), to ensure the inclusion of current regulatory frameworks and standard operating procedures. To ensure exhaustive coverage, the search strategy employed specific Boolean operators (AND, OR) to combine key terms across three thematic clusters. The exact search strings used were: (i) Context and Drivers: (“Antimicrobial Resistance” OR “AMR”) AND (“Food Chain” OR “One Health” OR “Animal Agriculture” OR “Aquaculture” OR “Environment”); (ii) Detection Technologies: (“Detection Methods” OR “Whole Genome Sequencing” OR “WGS” OR “Metagenomics” OR “CRISPR-Cas” OR “Biosensors”) AND (“AMR” OR “Pathogens”); and (iii) Mitigation Strategies: (“Biofilms” OR “Phage therapy” OR “Quorum sensing” OR “Food processing” OR “Biosecurity”) AND (“AMR mitigation” OR “Control”).

Regarding the temporal scope, the selection process prioritized studies published between 2010 and 2026, which constitute the majority of the cited literature, to reflect the most recent technological advancements in genomic surveillance (e.g., NGS, CRISPR-Cas) and current epidemiological trends. However, to provide necessary historical context regarding antimicrobial bans and the evolution of susceptibility testing standards, seminal papers and foundational regulations dating back to 2001 were also thoroughly reviewed. Following the screening and eligibility phases, a total of 204 peer-reviewed articles, official reports, and technical guidelines were included in the final qualitative synthesis.

The screening process involved an initial review of titles and abstracts to remove duplicates and irrelevant studies, followed by a full-text assessment based on predefined inclusion criteria. Specifically, the review selected peer-reviewed articles and official reports in English that addressed the transmission, detection, or control of AMR within the food production continuum. Studies focusing exclusively on clinical human treatments without a clear food safety or One Health link were excluded. To minimize bias, the literature screening and data extraction stages were conducted independently by three authors (E.G., P.S. and T.M.), with any discrepancies resolved through discussion or consultation with a fourth senior author (J.C.). A standardized data extraction form was utilized to systematically extract relevant information and ensure consistency across all included studies (provided as Supplementary Material Table S1). Data were synthesized qualitatively to identify key drivers of dissemination, compare the resolution of phenotypic versus genotypic methods, and evaluate integrated mitigation strategies, thereby supporting the review’s aim to identify and bridge existing knowledge gaps.

## 3. Sources and Drivers of AMR in the Food Chain

**Figure 2:**
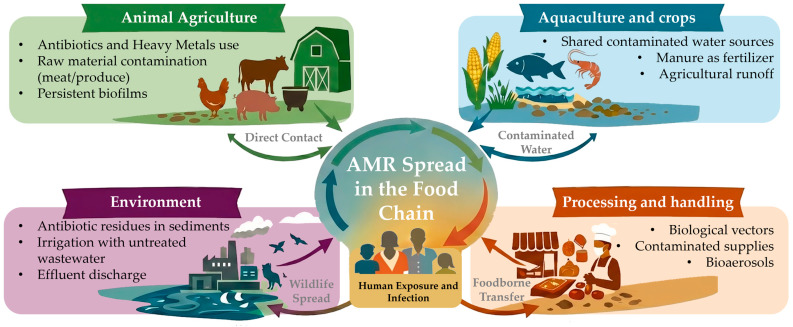
Schematic overview of interrelated pathways linking sources and drivers of AMR in animal, plant, environmental, and food-processing systems that interact and facilitate the spread of resistance throughout the food chain within a One Health context.

The food chain presents a complex and interrelated ecosystem where antimicrobial-resistant bacteria and resistance genes can emerge, persist, and spread, posing both direct and indirect risks to human health [5]. Therefore, understanding the key drivers and transmission routes of AMR in the food chain is essential to recognizing the factors contributing to this global threat and to develop effective mitigation strategies. In line with World Health Organization (WHO) recommendations, a coordinated One Health approach [15] is crucial for addressing AMR in a highly globalized food system, where resistance determinants can spread rapidly through trade, population mobility, and interconnected supply chains [16]. Without this multi-sectoral action, efforts to counter AMR risk become insufficient, with the potential to compromise the effectiveness of current antimicrobial therapies [17]. Accordingly, AMR in the food chain arises from multiple interconnected sources, with animal agriculture, aquaculture, crops, the environment, and food processing all playing significant and interdependent roles (Figure 2).

### 3.1. Animal Agriculture

Animal agriculture is a major source of AMR within the food chain due to the widespread use of antibiotics to promote growth and prevent disease in livestock, combined with the frequent occurrence of biofilms in animal housing and water distribution systems [5,18]. A substantial proportion of these antibiotics are administered at subtherapeutic doses, primarily to enhance growth performance and prevent infectious diseases [19]. Such practices apply strong and persistent selective pressure on animal-associated microbiomes, promoting the development and maintenance of multidrug-resistant pathogens, including vancomycin-resistant *Enterococcus* (VRE), carbapenem-resistant *Enterobacteriaceae* (CRE), and methicillin-resistant *Staphylococcus aureus* (MRSA) [20]. As such, the systematic integration of antibiotics into feed and drinking water, especially in poultry and swine production, further magnifies this selective pressure [19,20].

Although several high-income countries have implemented restrictions or prohibitions on the use of growth-promoting antibiotics, their use remains widespread in many LMICs, where over-the-counter access, limited veterinary supervision, and lack or insufficient regulatory surveillance contribute to high overall usage [16]. Beyond that, and even when antimicrobial drugs are used properly, resistant bacteria can still survive treatment and spread resistance determinants traits through spontaneous mutations or horizontal gene transfer via plasmids, transposons, and other mobile genetic elements [21].

In addition to antibiotic exposure, selective pressures in livestock environments can also arise from the use of heavy metals (such as copper and zinc) and biocides commonly incorporated into feed formulations and sanitation practices [22]. Importantly, many antimicrobial resistance genes are co-located with metal- and biocide-resistance determinants on common mobile genetic elements, implying that the exposure to these compounds can indirectly maintain and spread AMR even in the absence of such antibiotics [23]. This co-selection phenomenon further complicates mitigation strategies, as reducing antibiotic use alone may not be enough to stop antimicrobial-resistant bacteria from persisting and spreading in animal production systems. Moreover, microbial biofilms readily form in animal housing and water distribution systems, harboring pathogenic and antimicrobial-resistant bacteria that are protected from disinfection and that facilitate horizontal gene transfer, thereby enhancing the persistence and spread of resistance genes [18].

Therefore, AMR bacteria from animal agriculture can reach the human food chain through multiple ways. Direct transmission occurs through close contact with colonized animals, particularly among farmers and veterinarians [24]. There is also significant indirect exposure to resistant bacteria and genetic determinants contaminating meat, milk, and eggs during production and processing, entering domestic settings via retail food, or spreading through contaminated agricultural effluents [24]. Moreover, some studies showed that plasmid-mediated resistance genes can be transferred between foodborne pathogens and human-associated bacteria within food matrices [25,26]. Additionally, resistant bacteria and resistance genes from animal waste and biofilms can disseminate into soil and water, forming environmental reservoirs that reenter the food chain and pose risks to both animal and human health, being key routes for environmental spread [5]. Collectively, these routes increase the introduction of resistant strains into the human microbiome, thereby increasing the risk of infections that are difficult to treat [27].

### 3.2. Aquaculture and Crops

In parallel with animal agriculture and driven by exponential population growth and increasing pressure on limited planetary resources, there is a pressing need to rely on aquaculture and crop production to meet global food demand [6,28]. As a result, intensive aquaculture systems, characterized by high animal stocking densities, are known to cause physiological stress, making aquatic animals more susceptible to bacterial infections and thereby increasing the demand for antibacterial drugs [29]. As observed across other food-production sectors, the indiscriminate and widespread use of antibiotics to prevent disease and promote growth in fish and shellfish, particularly in regions with less stringent regulations, remains one of the most significant drivers of resistance selection, together with the accumulation of antimicrobial residues in sediments and surrounding waters [28], which frequently reach alarming concentrations that severely disrupt aquatic ecosystems and act as a continuous driver for resistance selection [30]. In general, production systems are strongly influenced not only by the prophylactic antimicrobial use but even more by metaphylactic practices, whereby entire livestock groups are treated with antibiotics in the water supply even when only a few animals show signs of infection [31]. Furthermore, bacteria in aquaculture environments quickly form biofilms on nets, tanks, and piping systems, enabling survival under antimicrobial pressure, persistence at high cell densities, and markedly increased rates of horizontal gene transfer [32].

Likewise, the use of pesticides and antimicrobial agents in plant agriculture, together with the application of contaminated water, manure, compost, and sewage sludge from livestock farms, can select resistance determinants and spread resistant microorganisms into crops [33]. In fact, the application of animal manure as a fertilizer may introduce antibiotic residues and multidrug-resistant bacteria coming from treated livestock [34]. Similarly, the use of contaminated surface water or wastewater, including sewage or farm runoff, for irrigation carries the same risk, since wastewater treatment facilities do not always completely remove antibiotic residues and microorganisms [35]. Consequently, selective pressure is imposed on the soil microbiomes, where the presence of low concentrations of antibiotics and heavy metals often found in manure promotes the survival and proliferation of resistant bacteria [36]. Furthermore, the soil environment is already rich in microorganisms, providing a natural setting for the horizontal transfer of genetic determinants of resistance between bacteria associated with plants and humans. Moreover, in contrast to animal-based products, which are usually cooked, many vegetables, such as leafy greens, are ingested raw. This means that resistant bacteria that persist on the surface are not destroyed during the cooking process and could potentially colonize the human gut [37].

Importantly, aquaculture and crop production systems are not isolated, but are inherently connected to surrounding ecosystems through the continuous discharge of effluents, runoff, and waste [27]. These biological exchanges establish the natural environment not merely as a passive recipient of contamination, but as a dynamic reservoir that actively drives the evolution and spread of AMR throughout the food chain [38].

### 3.3. Environment

While aquaculture and crop production systems act as major points of antimicrobial resistance selection and entry into the food chain, the broader environment represents an independent and highly dynamic reservoir where resistance determinants persist, evolve, and are redistributed across ecosystems and back into food production systems [39,40].

Environmental contamination from wastewater, and poor management practices, combined with selective pressures and gene transfer, are central to the emergence and spread of AMR [38].

Within a One Health framework, surface waters and wastewater treatment systems represent critical hotspots for AMR spreading through the environment, increasing their chance of reaching the food chain [41,42]. The occurrence and persistence of these antimicrobial drugs in aquatic ecosystems represent a severe environmental implication, acting as a continuous driver for resistance selection [30]. In practice, Wastewater Treatment Plants (WWTPs) receive all sorts of high-risk effluents, ranging from urban sewage to hospital-derived sewage [41]. In addition, the accumulation of non-antibiotic pollutants, such as heavy metals and biocides, exerts a strong selective pressure. As already noted, genes encoding resistance to metals and antibiotics are often located on the same mobile genetic elements or have common efflux mechanisms [43]. Consequently, environmental pollution can promote and spread AMR even in the absence of detectable antibiotic residues.

As conventional treatments often fail to eliminate resistance genes and chemical residues, biofilm rich WWTPs function as genetic mixing tanks, where environmental bacteria and human pathogens exchange genes before being released into the environment, such as rivers and other watercourses, thus facilitating gene exchange among bacteria and accelerating the spread of resistance [42]. Consequently, surface waters act as long-distance transport vectors, carrying these resistant bacteria from inland sources to coastal environments, where they can contaminate shellfish harvesting areas, or back into agricultural systems through irrigation, effectively recycling AMR back into the food chain [44].

Beyond hydrological pathways, environmental AMR spread also involves biological vectors, such as wildlife, particularly migratory birds, rodents, and insects. By feeding in contaminated areas, such as waste disposal landfills or near WWTPs, these animals can be colonized by resistant bacteria and physically transfer them to geographically distant regions or livestock farms that would otherwise be separated from such contamination [45].

Moreover, environmental AMR dynamics are strongly shaped by climatic and socio-economic factors. High temperatures have been correlated with increased bacterial growth rates and higher frequencies of horizontal gene transfer in aquatic systems, while heavy rainfall worsens contamination by causing sewer overflows and increasing agricultural runoff [46]. These factors are closely linked to socioeconomic conditions, as disparities in sanitation infrastructure and wastewater management directly influence the load of resistant bacteria being introduced into the ecosystem. Economic pressures in less regulated countries often prioritize high-yield production over biosecurity, fostering environments where resistance determinants spread due to underinvestment in pollution control and waste management [39,47,48].

However, AMR-related risks are not limited to pre-harvest environments. Once raw materials enter the industrial phase, additional selective pressures arise during processing and handling, further amplifying the potential for AMR spread.

### 3.4. Processing and Handling

By the time feedstock enters the industrial phase, the processing and handling environment presents a critical point where AMR can be intensified or further spread through specific vectors and structural niches. Food contact equipment and surfaces often serve as reservoirs for persistent bacterial colonization [49]. In these settings, bacteria readily form biofilms, which not only protect them from standard cleaning protocols but also promote cross-contamination between successive batches of meat or fresh products [50]. Beyond equipment, the supplies used for conservation and transport play a decisive role. Water and ice, often used for washing, cooling, or packaging, must be free of harmful microorganisms; otherwise, they act as efficient vehicles for spreading antimicrobial-resistant bacteria across large volumes of food products [51].

In addition, the processing environment itself is prone to contamination via poor air quality, dust, and the presence of vectors that can physically spread resistant strains throughout storage and preparation areas [45]. In this regard, the human element remains the most significant vector at this stage as food handlers can act as asymptomatic carriers of resistant *S. aureus*, *Escherichia coli*, and *Salmonella enterica*. Improper hygiene practices, such as insufficient hand washing or handling money during food service, facilitate the direct transfer of these pathogens from workers to ready-to-eat foods, avoiding any additional thermal elimination steps [52].

Nevertheless, the drivers of AMR in processing are not limited to handling contamination but also cover active selection pressures. Widespread use of biocides, disinfectants, and conservatives creates a chemically driven environment that can select for tolerant and resistant bacteria [53]. Long-term or sublethal exposure to these agents can promote cross-resistance to clinically important antimicrobials through common pathways such as multi-resistant efflux pumps [54]. Likewise, sublethal stresses from processing, including moderate heat, acidification, cold storage, or ultraviolet treatment, can have adverse consequences. When these preservation methods do not completely kill bacteria, the resulting stress response can trigger the expression of silent resistance genes or increase the rate of horizontal gene transfer, potentially enriching the final food product with highly resistant bacterial communities [55].

Ultimately, inadequate sanitation programs and the lack of rigorous control systems allow these bacteria to persist and recirculate within factories and households. As such, processing and handling should be considered not only as a logistical step, but as a key control point in mitigating the global AMR burden. In order to effectively break this cycle of transmission and ensure food safety, it is crucial to implement robust surveillance strategies that can quickly and accurately detect these resistant pathogens and their genetic determinants in the food matrix [56].

## 4. Detection of AMR in the Food Chain

The ability to detect and characterize AMR is a cornerstone of effective control and surveillance within the food chain [57]. In contrast to healthcare settings, where antimicrobial susceptibility testing informs patient therapy, AMR monitoring in food systems targets resistant bacteria and resistance determinants isolated from food-producing animals, food matrices, and associated production environments, without constituting clinical testing of foods themselves [5].

AMR emergence and dissemination occur across interconnected stages of the food chain, from primary production to consumption, reflecting complex ecological and evolutionary processes [5]. Addressing this complexity requires integrated surveillance strategies capable of capturing resistant microorganisms, resistance genes, and mobile genetic elements across heterogeneous food matrices and along the entire farm-to-fork continuum [40].

Current surveillance frameworks rely primarily on standardized phenotypic susceptibility testing of indicator and zoonotic bacterial isolates to ensure regulatory harmonization and longitudinal comparability. These approaches are increasingly complemented by high-resolution molecular and genomic methodologies, which enable detailed characterization of resistance mechanisms, evolutionary trajectories, and transmission dynamics within and across food systems [40,58]. This section critically evaluates these complementary methodologies, with particular emphasis on their analytical resolution, applicability across food systems, and current limitations, in the context of strengthening global AMR surveillance and ensuring food safety [59].

### 4.1. Conventional Methods

Conventional culture-based methods remain the gold standard for AMR detection in food surveillance systems worldwide [60,61]. These methods rely on the isolation of viable bacteria from food matrices followed by phenotypic testing to determine susceptibility to specific antimicrobial agents [62,63]. Their widespread adoption is driven by their reproducibility and standardized protocols, and their results provide clinical relevance since the phenotypic resistance confirms that a bacteria can survive while being exposed to a given antibiotic [8,60].

#### 4.1.1. Sample Preparation and Isolation

The reliability of any conventional method begins with sample preparation, a critical step regulated by international standards such as ISO 6887 [64]. Different food matrices pose significant and distinct challenges regarding bacteria recovery [65,66]. Standard protocols require the homogenization of samples to release bacteria from the matrices without compromising their viability [64].

Following homogenization, the isolation of target pathogens, such as *Salmonella* spp., *Campylobacter* spp., and *E. coli*, typically involves a multi-step workflow [67]. A non-selective pre-enrichment step allows for the resuscitation of injured cells, which are common in food matrices due to cold and heat stress, or exposure to food preservatives and other chemicals. Buffered Peptone Water (BPW) is the standard medium used in this step, which is typically incubated at 37 °C [64,68]. This step is followed by the selective enrichment, in which samples are transferred to specialized media containing inhibitory substances that suppress competing microbiota while favoring the growth of the target microorganism [69]. Then selective and/or differential plating is employed where the enriched culture is streaked onto selective agar plates to ensure the isolation of the target pathogen [70]. Also, selective and/or differential media can be used for AMR surveillance, and specific protocols have been developed to screen directly for resistant phenotypes, as the case of Extended-Spectrum Beta-Lactamase (ESBL), *AmpC* beta-lactamase, and Carbapenemase-producing *E. coli* [71]. The European Union Reference Laboratory for Antimicrobial Resistance (EURL-AR) recommends that protocols use MacConkey agar supplemented with cefotaxime for the selective isolation of ESBL/AmpC producers [71,72]. This allows resistant populations to be recovered that might otherwise be outgrown by susceptible bacteria on non-selective media. Furthermore, chromogenic agar media have revolutionized this stage by incorporating chromogenic substrates with antibiotics that are hydrolyzed by specific bacterial enzymes, resulting in different colored colonies [70]. This allows a rapid visual differentiation of target resistant pathogens, such as MRSA or VRE, directly at the plate [70,73].

#### 4.1.2. Phenotypic Antimicrobial Susceptibility Testing (AST)

After obtaining a pure culture, Antimicrobial Susceptibility Testing (AST) can be performed to evaluate resistance, using the methods described below (Table 1).

The Broth Microdilution (BMD) method is currently the internationally accepted reference method for AST in food surveillance, as defined by ISO 20776-1 and CLSI M07 standards [74,75], and is highly suitable and cost-effective for rapidly growing, non-fastidious foodborne pathogens like *Salmonella enterica* and *Escherichia coli*. In this method, bacteria are inoculated into a series of wells containing Mueller-Hinton broth with known concentrations of different antibiotics [9]. Then, the Minimum Inhibitory Concentration (MIC) is determined as the lowest concentration that inhibits visible growth, when no turbidity is observed [76]. This technique provides a quantitative result (MIC value), rather than a qualitative result. Additionally, it is a very standardized technique since the use of automated pipetting and commercially available 96-well plates make it easy to perform and harmonize across reference laboratories [63].

While the Disk Diffusion (Kirby–Bauer) method is less common in more advanced laboratories, it remains widely used in clinical and smaller food testing laboratories due to its low cost and simplicity [77]. In this approach, antibiotic disks are placed on an agar plate inoculated with a bacterial lawn, and after 24 h of incubation, the diameter of the inhibition zone is measured and interpreted as susceptible or resistant [78]. Although effective for initial screening, this method does not provide precise MIC values, thereby limiting its utility for quantitative risk assessment in the food chain [79].

The Gradient Strip Method (E-test) employs plastic strips loaded with a predefined antibiotic concentration [76]. It combines the simplicity of diffusion with the quantitative capability of MIC determination [76]. It is useful for confirming ambiguous results from automated systems or for testing fastidious organisms like *Campylobacter* spp., for which broth dilution can be technically demanding [80].

#### 4.1.3. Regulatory Frameworks

An important aspect of AMR testing and surveillance is the interpretation of the data obtained. The classification of an isolate as “Resistant” or “Susceptible” depends on the breakpoints applied [81]. Two major institutions set these standards: CLSI (Clinical and Laboratory Standards Institute), dominant in the USA and many international settings [82], and EUCAST (European Committee on Antimicrobial Susceptibility Testing), widely adopted in Europe and increasingly globally [83].

Additionally, a major difference in food safety when compared to clinical diagnostics is the use of Epidemiological Cut-off Values (ECOFFs), instead of Clinical Breakpoints [84]. Clinical Breakpoints predict the likelihood of therapeutic success in treating a patient [81], while ECOFFs distinguish the “wild-type” population (without acquired resistance mechanisms) from the resistant population [85]. For food chain surveillance, EFSA and EURL-AR prioritize ECOFFs because they are more sensitive in detecting the emergence of low-level resistance that may not yet result in clinical failure but represents a significant transmission risk [63].

### 4.2. Molecular Approaches

While conventional methods describe the phenotype, molecular approaches target the genotype. The recent advances in molecular diagnostics for the food chain are driven by the need for fast results, the ability to detect non-culturable organisms, and the requirement to trace transmission pathways [86]. These methods focus on the detection of specific Antimicrobial Resistance Genes (ARGs) or point mutations associated with resistance [87].

**Figure 3:**
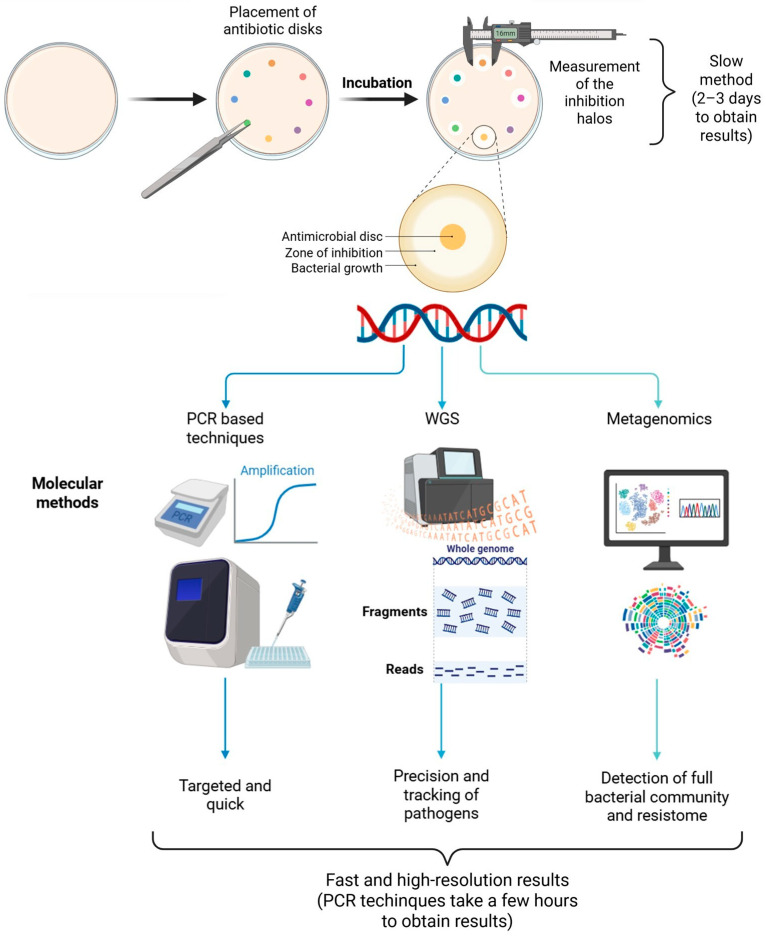
Comparative overview of traditional versus molecular approaches for AMR detection in the food chain. The figure illustrates the workflow differences between conventional phenotypic methods (**top**), characterized by culture isolation and longer time-to-result, and genotypic methods (**bottom**), including PCR, Whole Genome Sequencing (WGS), and metagenomics, which offer rapid detection and high-resolution genetic characterization.

The comparison between these molecular approaches and regulatory phenotypic methods is summarized in Figure 3.

#### 4.2.1. Polymerase Chain Reaction (PCR)-Based Methods

PCR remains the main tool for molecular detection due to its specificity and sensitivity [88]. It amplifies targeted DNA sequences, enabling the detection of known resistance genes [89]. Multiple variants of the conventional PCR technique are currently used in AMR surveillance, the most relevant of which are described below.

Multiplex PCR enables multiple target genes to be amplified simultaneously in a single reaction using different primer pairs [67]. This method increases the speed of the results and reduces reagent costs compared to single-target assays, making it ideal for large-scale screening [67]. This technique is valuable for screening complex food matrices where diverse resistance mechanisms and bacterial species often co-exist [90]. However, implementing multiplex PCR is technically challenging as it requires rigorous optimization to ensure compatible annealing temperatures for all primer sets and prevent the formation of primer-dimers, which can inhibit the reaction [91]. Furthermore, competition for shared reagents, such as DNA polymerase and dNTPs, can lead to preferential amplification of abundant targets, potentially masking low-abundance resistance genes. Careful validation of limit-of-detection (LOD) is therefore required for each target [92].

Real-Time Quantitative PCR (qPCR) outperforms traditional PCR by monitoring amplification in real-time using fluorescent markers, enabling the precise quantification of ARG copy numbers using a calibration curve rather than just their presence/absence detection [92]. This capability represents a major advantage for food safety applications, as ARG abundance may correlate more closely with the potential for horizontal gene transfer than qualitative data alone [40]. There are two main ways this technique can be employed, using non-specific DNA-binding dyes like SYBR Green, which are cost-effective but may bind to non-target double-stranded DNA (including primer-dimers), and sequence-specific hydrolysis probes (such as TaqMan), which offer superior specificity and allow for multiplexing within the qPCR format by using different fluorophores [93]. Despite its sensitivity, qPCR in food analysis faces the challenge of matrix-derived inhibitors [94]. Certain substances such as calcium ions in milk, humic acids in vegetables, and heme groups or hemoglobin in meat products can inhibit DNA polymerase activity, leading to reduced ARG results or false negatives [94]. Therefore, the inclusion of internal amplification controls is mandatory to validate reaction efficiency in these complex matrices [95].

Viability PCR (v-PCR) tackles a major limitation of the conventional PCR and qPCR, which is the inability to distinguish between DNA from viable bacteria from the remaining DNA from dead cells, that can lead to false-positive cases in foods that have undergone heat treatment or disinfection [96]. Viability PCR addresses this by using DNA-intercalating dyes such as Propidium Monoazide (PMA) or the more recent PMAxx, which offers enhanced selectivity [97,98]. These dyes are added to the sample prior to DNA extraction [97]. They penetrate only the compromised membranes of dead cells and, upon photo-activation with high-intensity light, covalently bind to DNA [97]. This modification physically blocks the DNA polymerase, preventing amplification of “dead” DNA during the PCR [97]. v-PCR has been successfully applied to detect viable *Salmonella*, *Listeria*, and *Campylobacter* in matrices like lettuce, milk, and poultry [99,100,101]. However, this technique relies on light penetration; therefore, more turbid samples can inhibit the effect of the treatment with the dyes, leading to incomplete suppression of the amplification of DNA from dead cells [102]. Additionally, older dyes like Ethidium Monoazide (EMA) have been shown to be toxic to some viable bacterial species (e.g., *Campylobacter*), potentially causing false negative results [103].

#### 4.2.2. Whole Genome Sequencing (WGS)

WGS has altered the landscape of AMR surveillance, moving from a research tool to the new reference standard for regulatory agencies like the FDA (GenomeTrakr) and EFSA [86].

Most routine regulatory surveillance currently relies on short-read sequencing (such as Illumina), which generates highly accurate reads of small DNA fragments (typically 150–300 bp) [104]. While this technology is cost-effective and the industry standard for SNP-based phylogenetic typing, it has a significant limitation in AMR analysis: it struggles to resolve repetitive DNA regions, which are common in plasmids and transposons where resistance genes be located in [105]. As a result, short reads often fail to bridge these repeats, resulting in fragmented genome assemblies where it is impossible to determine if an ARG is located on the chromosome (vertical transmission) or on a mobile plasmid (horizontal transmission) [105]. To overcome this limitation, long-read sequencing technologies, like Oxford Nanopore Technologies and PacBio, have emerged, capable of sequencing single molecules with thousands of base pairs in length [106]. Although individual long reads exhibit lower base-level accuracy, these long reads can detect repetitive regions, allowing for the complete assembly of circular plasmids [107]. The integration of both technologies, called Hybrid Assembly, is increasingly becoming the gold standard for characterizing the mobile genetic elements present in foodborne pathogens, allowing them to definitively track the movement of multidrug-resistance plasmids between different bacterial species in the food chain [107].

Using WGS data, phenotypic resistance profiles can be predicted by comparing the sequenced genome with curated databases, such as ResFinder [108]. One key metric used to evaluate the adoption of this technology is the Genotype-Phenotype Correlation, which estimates the degree to which the presence of a gene predicts the actual resistance profile [109]. Extensive validation studies demonstrated very high concordance rates (often exceeding 95%) for key foodborne pathogens like *Salmonella* against clinically relevant antibiotics, whereas predictive accuracy drops significantly (below 80%) for species with complex, multifactorial resistance mechanisms like *Pseudomonas* spp. [109]. This reliability has led national surveillance programs to rely less on routine phenotypic testing and more on WGS-only workflows [110]. However, there are some risks associated with this technology, such as certain complex resistance mechanisms (e.g., specific aminoglycoside modifications) or novel resistance genes that are not yet cataloged in databases can lead to false-negative predictions [87].

Beyond resistance detection, WGS enables source attribution, a method used to identify the origin of infections and outbreaks [111]. By analyzing the core genome, which are shared essential genes, and calculating SNP distances, epidemiologists can match bacterial strains with high precision [112]. This capability allows public health agencies to determine which specific food sources are responsible for antibiotic-resistant infections [113]. Consequently, surveillance shifts from passive monitoring to proactive intervention, enabling regulators and agencies to pinpoint the exact region or food product causing issues and implementing the required mitigation strategies [114].

#### 4.2.3. Metagenomics

While WGS requires a pure culture, metagenomics sequences all DNA present in a sample, being capable of providing a culture-independent analysis of the entire microbial community and its collection of resistance genes [115].

The shotgun approach fragments all DNA in a sample and sequences it randomly [116]. This provides information about all ARGs present, including those in non-pathogenic organisms such as commensal species or environmental bacteria, which often serve as reservoirs for resistance [116,117]. One of its main advantages is that it can detect the full spectrum of resistance potential in a food product or production environment, and not only in the bacteria that would grow in a culture plate [116]. However, a significant drawback is that the majority of DNA in a food sample comes from the host animal, rather than the bacteria present in it [118]. Therefore, techniques to remove host DNA are crucial, these include differential lysis (lysing host cells while keeping bacteria intact) or the use of specific commercial kits that enzymatically degrade non-bacterial DNA prior to extraction [119].

Another limitation of traditional short-read metagenomics is the loss of context. While it can detect a resistance gene and a pathogen in the same sample, it is difficult to attribute genes to pathogens [105]. Even so, the inability to link plasmids to their host bacteria makes risk assessment difficult [105]. To address this challenge, long-read metagenomics bridges the gap between resistance genes and their hosts by sequencing them together on long DNA fragments [120]. Furthermore, advanced bioinformatic tools can now exploit DNA methylation patterns to associate plasmids with their specific bacterial hosts [121]. Together, these advances enable the culture-free identification of resistant carriers in complex food matrices.

Metagenomics has also a strong application in the One Health surveillance of food production environments [122]. It is increasingly being used to monitor the resistome in wastewater and soil in agricultural settings, providing insight into environmental reservoirs of resistance [123,124]. Unlike the conventional PCR, which targets predefined genes only, metagenomics offers a complete and comprehensive view of microbial community composition and resistance dynamics [116]. By detecting resistance trends and novel genes before their emergence in clinical settings, this approach is important to active ecosystem monitoring [125].

### 4.3. Rapid and Emerging Technologies

The necessary time to obtain results from conventional methods (2–4 days) and even sequencing-based approaches (days to weeks) is often incompatible with the short shelf-life of many food products [67]. For instance, while a phenotypic AST requires 48–72 h to yield results, targeted qPCR can provide data in under 4 h, and nanopore sequencing can generate actionable resistome profiles within 6 to 12 h. Consequently, there is increasing interest in technologies that offer real-time or near real-time detection, including Point-of-Care (POC) capabilities [126].

#### 4.3.1. MALDI-TOF MS

Matrix-Assisted Laser Desorption/Ionization Time-of-Flight Mass Spectrometry (MALDI-TOF MS) analyzes the protein mass spectrum of a bacteria [127]. In this technique, bacterial colonies are mixed with a matrix, ionized by a laser, and the time it takes for the ions to travel creates a characteristic spectrum specific to each bacterium [127].

Beyond species identification, MALDI-TOF is increasingly used to explore AMR detection [128]. This can be achieved by detecting specific resistance-associated proteins or by observing the antibiotic hydrolysis [128]. For example, beta-lactamase assays involve incubating bacteria with a beta-lactam antibiotic, such as carbapenem, for a few hours and followed by detection of mass shifts corresponding to hydrolyzed antibiotic molecules [128].

One of the main advantages of MALDI-TOD MS is that species identification can be achieved within minutes once a colony is available [129]. However, it still relies on prior culture isolation, meaning that it speeds up the species confirmation but does not eliminate the need for initial culture isolation [129].

#### 4.3.2. Nucleic Acid-Based Biosensors and Isothermal Amplification

The rapid and accurate detection of AMR is essential for ensuring food safety, monitoring environmental reservoirs and enabling timely interventions. When combined with isothermal amplification techniques such as Loop-Mediated Isothermal Amplification (LAMP) and Recombinase Polymerase Amplification (RPA), nucleic acid-based biosensors offer a highly sensitive, specific and portable approach to AMR surveillance. Unlike conventional PCR, these methods operate at a constant temperature, eliminating the need for thermocycling equipment and enabling deployment in the field or in settings with limited resources [130].

Several studies have demonstrated the versatility and efficiency of these platforms. For instance, LAMP-based assays have successfully detected various AMR genes, such as *mcr-1*, *blaKPC*, and *blaOXA* variants, in pond water samples within approximately one hour, demonstrating the potential for rapid environmental monitoring [131].

Advances in electronic biosensors have further expanded the range of applications for isothermal amplification. Thin-film transistor (TFT) sensors coupled with RPA have demonstrated the ultra-fast detection of β-lactam and carbapenem resistance genes, such as CTX-M and NDM, in *E. coli* and *K. pneumoniae* isolates [132]. This provides real-time, quantitative results within minutes. These examples collectively illustrate how nucleic acid-based biosensors can be adapted to various sample matrices, including environmental water, food products and clinical isolates, while maintaining rapid turnaround times, portability and high sensitivity.

#### 4.3.3. CRISPR-Cas Diagnostics

The adaptive immune system of bacteria, CRISPR-Cas, has been repurposed for molecular diagnostics [133,134,135]. Platforms such as SHERLOCK (Cas13) and DETECTR (Cas12a) use programmable guide RNAs to recognize specific nucleic acid sequences, including genes associated with antimicrobial resistance [133,134]. When the Cas enzyme binds to the target ARG, it cleaves nearby reporter molecules, releasing a detectable fluorescent signal [134].

These assays can achieve sensitivities comparable to PCR while operating under isothermal conditions, thereby eliminating the need for thermocyclers and enabling deployment on simple paper-based formats or portable devices [135]. For instance, Cas12a-based assays have been successfully applied to detect the *bla*_KPC-2_ gene, which confers resistance to β-lactam antibiotics, demonstrating high specificity and operational simplicity and highlighting their strong potential for rapid antimicrobial resistance screening in clinical and field settings [136].

### 4.4. Detection Gaps

Despite these major technological advances, significant gaps remain that make it difficult to establish a comprehensive AMR surveillance network across all food chain [137].

#### 4.4.1. The Viable but Non-Culturable (VBNC) Blind Spot

A major limitation of the gold standard culture methods is the lack of capacity to detect bacteria in the VBNC state [138]. Different food processing stages often induce this state, where bacteria remain metabolically active and potentially virulent, but fail to grow on standard agar plates [139]. Consequently, food products can appear to be safe by regulatory standards but still have pathogenic resistant bacteria that resuscitate after being ingested [140]. While qPCR and metagenomics can detect these, they lack regulatory methods, creating some disparities between legal safety testing and actual risk [141].

#### 4.4.2. Matrix Interference and Sample Complexity

Food matrices are chemically complex and often contain potent inhibitors of PCR polymerases, such as calcium in milk and heme groups in meat [94] that are compounds that can chelate Mg^2+^ ions or interact directly with DNA, leading to false-negative results in molecular assays [94].

Additionally, it is impossible to develop a universal extraction protocol that eliminates these inhibitors; methods optimized for certain types of matrices that can fail when used with different types of matrices [94]. The lack of standardized sample preparation protocols for molecular methods introduces inter-laboratory variability and limits data comparability, complicating AMR surveillance efforts.

#### 4.4.3. The Genotype-Phenotype Disconnect

Molecular methods detect the presence of a certain gene, not its expression. An isolate may harbor an ARG that is transcriptionally silent due to promoter defects or regulatory mutations, resulting in a phenotypically susceptible profile [142]. Additionally, new resistance mechanisms (such as efflux pump overexpression or porin loss and mutations) may not yet be captured by WGS databases, leading to false susceptible genotype predictions [143]. This gap underscores the continued use of phenotypic testing to confirm the results obtained by genotype surveillance, preventing a genomic-only surveillance to be implemented [144].

#### 4.4.4. Global Standardization Challenges

While the EU and USA are moving towards WGS-based surveillance, this technology remains too expensive and logistically inaccessible for many LMICs, which often bear the highest burden of foodborne AMR and its emergence is most critical [144]. Implementing WGS requires not only sequencers but also robust bioinformatic infrastructure, data storage, and skilled personnel, all of which are often lacking in these regions [145].

Even among High-Income Countries (HICs), the lack of harmonized sequencing protocols, analytical pipelines, and reporting standards limits the comparability of genomic and metagenomic data across borders, hindering global surveillance and coordinated responses [145].

#### 4.4.5. Fragmentation of the One Health System

Current AMR surveillance remains highly fragmented, since data from veterinary, food, and human settings are kept in separate databases, which are managed by different agencies with different rules and standards [146]. Bridging these gaps requires not just technological solutions, but political and administrative solutions to create data platforms that allow resistant genes to be tracked from farm to fork to patient within a unified One Health framework [146].

Therefore, the effective AMR detection in the food chain currently relies on a hybrid surveillance approach. The conventional methods provide phenotypic information for regulatory standards, while molecular and emerging technologies offer the speed and resolution required for a quick trace-back of the pathogens, as it is described in Table 2 [147]. Addressing existing gaps will require efforts to standardize molecular protocols, reduce costs for global implementation, and integrate all the data into a single One Health system [147].

The analysis of current AMR detection reveals that a transition is happening, since it is moving from a system based on culturing pathogens after contamination has occurred, towards a system based on genomics [122]. However, this transition faces challenges that extend beyond simple technical limitations [122].

The first major issue is the discrepancy between data generation and results. While modern sequencing generates massive amounts of data, food safety officers do not need this complexity, they require risk indicators to make decisions [148,149]. Currently, the lack of user-friendly decision-support tools translating genomic data into regulatory actions limits practical implementation [149]. Additionally, diagnostic innovation is evolving faster than the regulatory framework, while new diagnostic tools can detect contamination in minutes, legislation often still mandates slow culture-based confirmation, undermining the utility of rapid diagnostics [150]. Accelerated validation and regulatory acceptance of molecular methods are therefore essential [150].

Finally, current AMR surveillance often misses the mobile genetic elements that spread resistance [151]. Antimicrobial resistance is driven by plasmids capable of transferring between species, meaning that a harmless commensal bacterium can harbor a resistance gene and subsequently pass it to a pathogenic one [151]. This process is particularly favored in environmental biofilms, where high cell density and close cell–cell contact strongly enhance horizontal gene transfer mechanisms [152]. Future strategies must monitor the potential of transmission of these genetic elements, rather than just the bacteria themselves [151]. Given the globalized nature of the food supply chain, equitable access to advanced detection technologies is critical, as resistance emerging in resource-limited settings can rapidly spread worldwide [153].

## 5. Mitigation Strategies Across the Food Chain

Efforts to combat AMR must not be limited to isolated interventions within the food chain. Instead, mitigation requires a coordinated and multistep approach that addresses the causes of resistance at all stages, including production, processing, distribution, and consumption. Therefore, control measures must span the entire food chain, from farm to fork, as it is a significant route for the persistence and dissemination of resistant bacteria and resistance genes. However, the precise magnitude of this spread has not yet been fully quantified. Effective AMR detection and control across all stages require interprofessional collaboration, robust regulatory frameworks, expanded surveillance systems, and continuous stakeholder training.

### 5.1. On-Farm

On-farm mitigation strategies represent the frontline of defense in preventing the emergence and dissemination of AMR within the food chain. These interventions must address the core driver of the problem, namely antimicrobial misuse. As the farming sector serves as a critical interface between antimicrobial pressure, bacterial populations, animal health, and the environment, a comprehensive and well-integrated set of management measures is required.

#### 5.1.1. Prudent Antimicrobial Stewardship

Prudent antimicrobial stewardship is a well-documented approach that has been shown to be effective in reducing resistance in both animal and human populations, often without compromising farm productivity. Studies confirmed that interventions aimed at restricting antibiotic use in food-producing animals lead to a significant reduction in the prevalence of antibiotic-resistant bacteria in livestock [154]. Crucially, this reduction extends beyond the farm, with parallel decreases observed in resistant bacteria within human populations, highlighting the direct link between on-farm source control and public health safety [154].

Several European countries have successfully implemented evidence-based stewardship programs grounded in scientific evidence. A notable example is the Danish ban on avoparcin, a structural analog of vancomycin previously used as a growth promoter [155]. This measure led to a drastic decline in VRE in both livestock and human populations within two years [155]. Similarly, Australia has maintained strict regulatory policies regarding quinolone use in food-producing animals [156]. Consequently, resistance rates to this antimicrobial class in pathogens such as *E. coli* and *Campylobacter* spp. remain significantly lower than in countries with less restrictive policies.

#### 5.1.2. Enhanced Biosecurity and Farm Management

Biosecurity and farm management practices constitute fundamental pillars of on-farm AMR mitigation [157]. Studies on biosecurity and water, sanitation, and hygiene (WASH) interventions highlight the efficacy of these measures [157]. Literature consistently demonstrates that improved farm management leads to a significant reduction in the reliance on antimicrobials, while manure-targeted interventions can drastically decrease the load of ARGs and resistant bacteria in animal waste [157]. Consequently, there is a strong consensus linking the implementation of high biosecurity standards with a marked reduction in antimicrobial consumption [157].

Key biosecurity measures that are essential for breaking transmission cycles include the compartmentalization and segregation of animal groups, alongside “all-in/all-out” production systems. These practices must be supported by rigorous hygiene protocols, encompassing regular cleaning and disinfection of facilities, equipment, and contact surfaces [158]. Additional critical components include safe feed and water management to prevent pathogenic contamination, continuous personnel training to ensure compliance, and meticulous and systematic record-keeping to track antimicrobial use and monitor resistance trends [158].

#### 5.1.3. Alternative Disease Prevention and Control Strategies

Beyond stewardship and biosecurity, alternative disease prevention strategies are increasingly recognized as indispensable components of a comprehensive on-farm AMR mitigation framework. These include vaccination programs as well as use of probiotics and prebiotics, organic acids, antimicrobial peptides, and plant-derived compounds, which act through complementary mechanisms to enhance host immunity, modulate the gut microbiota and control microorganism colonization. These interventions aim to maintain animal health and productivity while minimizing reliance on conventional antimicrobials. A summary of the principal alternative strategies and their mechanisms is provided in Table 3.

### 5.2. Processing & Retail

Mitigating AMR at the processing and retail stages requires the strict implementation of hygiene protocols, effective biofilm control measures, and robust surveillance systems to prevent the persistence and dissemination of resistant bacteria and ARGs through food chain. Food processing environments constitute Critical Control Points (CCPs), where high bacterial loads can be rapidly disseminated if adequate sanitation and safety protocols are compromised [171].

#### 5.2.1. Innovative Biofilm Control Approaches

**Figure 4:**
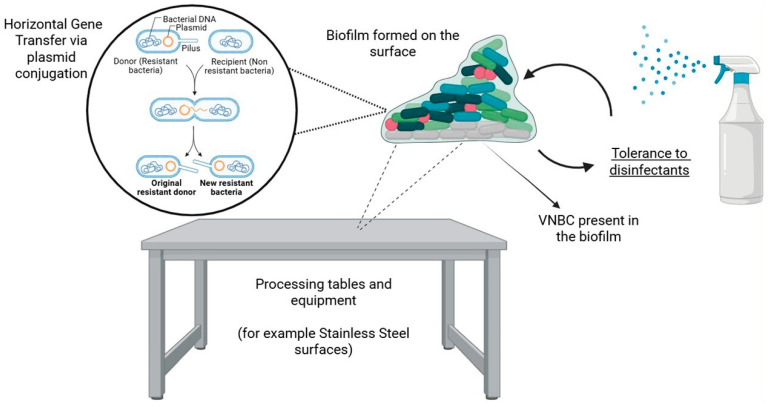
Mechanisms of antimicrobial resistance and persistence within food processing biofilms. The illustration shows a multispecies bacterial community adhered to a stainless-steel surface, embedded in a protective extracellular polymeric substance (EPS) matrix. Key resistance features include physical tolerance, where the EPS matrix acts as a barrier hindering the penetration of disinfectants and sanitizers, the presence of viable but non-culturable (VBNC) cells that survive antimicrobial treatment and are metabolically active, and enhanced Horizontal Gene Transfer (HGT) of resistance plasmids between bacteria within the biofilm structure.

Biofilm-mediated AMR represents an often-underestimated but highly persistent challenge in food safety [172]. These structured microbial communities function as protective reservoirs, significantly enhancing bacterial survival and tolerance to antimicrobials and disinfectants, as illustrated in Figure 4 [173]. Consequently, biofilms constitute a critical issue for the food and other industries, since this protective mode of growth allows foodborne pathogens to withstand processing stressors, leading to persistent contamination of equipment and posing a significant risk to public health risks [174].

To overcome the limitations of conventional sanitation practices, innovative biofilm control strategies increasingly focus on preventing initial attachment and disrupting established biofilm communities through surface engineering and biological control interventions. Surface modification and antimicrobial coatings modify the surface properties of food processing materials, such as stainless steel and plastics, and represent effective approaches to reduce bacterial colonization [175,176]. These strategies include coating deposition, surface texturing, and chemical treatments [175,176].

These interventions operate through three primary mechanisms, more exactly: (1) decreasing surface roughness to minimize available attachment sites for bacterial adhesion, (2) introducing antimicrobial agents directly onto the surface to actively inhibit growth, (3) and applying hydrophobic coatings to repel water and organic matter, thereby impeding the initial stages of biofilm formation [175,176,177,178].

Quorum sensing (QS) inhibition and plant-derived compounds represent additional promising biofilm control strategies. Certain essential oils derived from medicinal plants exhibit potent anti-biofilm and anti-QS activities [169]. Mechanistically, these compounds down-regulate genes involved in QS signaling (such as *luxR*, *luxS*, *qseB*, and *sdiA*) and biofilm structural components (such as *csgA*, *csgB*, *csgD*, *flhD*, *fliZ*, and *motB*) [179,180]. Among evaluated compounds, rose, geranium, lavender, and rosemary oils have demonstrated superior inhibition activity [169].

A synergistic approach combining QS inhibitors with exopolysaccharide (EPS) degraders not only suppresses microbial growth but also enhances the efficacy of cleaning protocols [181]. This strategy reduces contamination risks, thereby extending the shelf life and safety of food products. Importantly, plant-based biofilm control compounds offer a sustainable alternative that does not contribute to the development of chemical or antimicrobial resistance [170,181,182,183].

Finally, bacteriophage-based biocontrol represents a highly specific biological strategy for eliminating bacterial pathogens without disrupting the commensal microbiota [184]. Phages are viruses that specifically infect and lyse target bacteria while remaining harmless to mammalian cells [184]. This host specificity offers a distinct advantage over broad-spectrum antimicrobial agents, ensuring pathogen eradication while preserving beneficial microbial balance [185,186,187].

#### 5.2.2. Stringent Hygiene and Sanitation Protocols

Rigorous implementation of comprehensive sanitation protocols [188] constitutes the cornerstone of AMR mitigation in food processing environments. To effectively prevent biofilm formation and reduce the dissemination of resistance, protocols must encompass five key dimensions: (1) thorough pre-cleaning to eliminate organic matter that serves as a substrate for biofilm development; (2) chemical sanitization using approved agents at optimal concentrations and contact times; (3) routine verification testing to confirm sanitation efficacy; (4) surveillance systems capable of detecting persistent contamination; and (5) meticulous documentation to ensure full traceability of all sanitation activities [189,190].

#### 5.2.3. Surveillance and Monitoring Systems

Beyond traditional methods, critical innovations in hygiene management emphasize the integration of rapid verification technologies. Tools such as ATP bioluminescence monitoring, real-time microbial detection systems, and advanced molecular assays (qPCR and digital PCR) can enhance sanitation effectiveness, and this shift facilitates a transition from reactive corrective actions to proactive and risk-based hygiene management [191].

The integration of high-throughput molecular methods into routine surveillance systems is crucial for proactive AMR risk management [192]. While conventional culture-based methods remain the legal gold standard, their 48–72 h turnaround time often delays critical decision-making [9]. In contrast, the adoption of rapid platforms like qPCR, biosensors, and on-site molecular diagnostics allows the generation of same-day actionable data [193,194,195]. This shift enables processors to quarantine or treat contaminated batches before they enter the retail chain, thereby preventing recalls and ensuring consumer safety.

### 5.3. Consumer Level

Mitigating AMR spread at the consumer level is a critical, yet a frequently overlooked component of the food chain strategy. Contaminated food products constitute a direct vector for human exposure to antimicrobial-resistant bacteria and ARGs [5]. Consequently, consumer behavior acts as the last line of defense in interrupting transmission cycles. Despite the implementation of strict surveillance at the farm and processing stages, improper domestic practices, such as cross-contamination between raw and cooked foods, inadequate thermal processing, and poor hygiene, can negate upstream efforts, facilitating the colonization of the human gut by resistant pathogens [196,197,198].

#### Food Handling and Hygiene Practice

Studies indicate a high prevalence of antimicrobial-resistant bacteria in retail meat and seafood, with some reports showing median prevalence rates exceeding 50% for pathogens such as *Campylobacter*, *Enterococcus*, *Salmonella*, *E. coli*, *Listeria*, and *Vibrio* spp. [186,199,200,201]. This scenario underscores the significant exposure risk consumers face through the handling and consumption of contaminated raw products.

The “4Cs” of food safety, more exactly, Cleaning, Cooking, Chilling, and avoiding Cross-contamination, remain the cornerstone measures for reducing AMR transmission risks [202]. Adequate thermal processing is particularly critical, as proper heat treatment effectively eliminates viable bacteria, including resistant strains, rendering the food safe for consumption [203]. However, consumer awareness and strict compliance with these hygiene practices vary widely across different demographic groups, reducing their overall effectiveness [203].

Given the ability of resistant bacteria to persist in domestic environments, preventing cross-contamination is crucial. Evidence suggests that contaminated kitchen surfaces often act as reservoirs for resistant pathogens, facilitating their transfer to ready-to-eat foods during preparation [204]. Consequently, the strict separation of raw and cooked foods, thorough disinfection of food-contact surfaces, and proper hand hygiene are therefore key interventions to mitigate domestic AMR spread [196,204].

**Figure 5:**
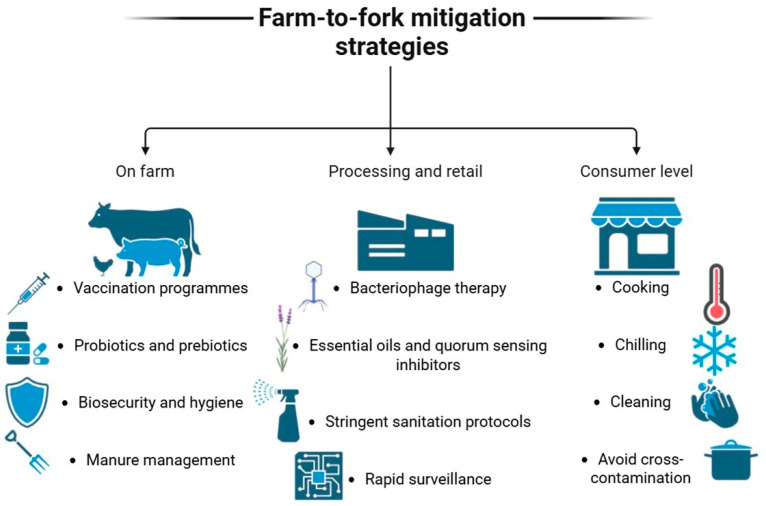
Integrated mitigation strategies to combat antimicrobial resistance across the food chain (“Farm-to-Fork”). On-farm strategies include the implementation of biosecurity measures, manure management, and alternative therapies such as vaccination, probiotics and strict hygiene protocols. At the processing and retail level, the focus is on biofilm control using novel quorum sensing inhibitors (such as essential oils), bacteriophages, and rigorous sanitation protocols supported by rapid monitoring. Lastly, at the Consumer Level, an adherence to the “4Cs” of food safety: proper Cooking (thermal elimination), Chilling, Cleaning (hand hygiene), and preventing Cross-contamination between raw and cooked foods.

A summary of mitigation strategies across the food chain is presented in Figure 5.

## 6. Conclusions

The dissemination of AMR within the food chain poses a serious and escalating threat to global public health and food security, requiring a shift from reactive control to proactive and integrated surveillance. This review highlights that, while the food chain acts as a vast, interconnected reservoir for resistant bacteria and resistance genes, our ability to detect, interpret, and respond to these threats is currently undergoing a critical transition.

Moving from traditional culture-based methods to advanced genomic technologies, such as WGS and metagenomics, offers unprecedented resolution for tracking transmission pathways and characterizing resistance mechanisms. However, there is still a significant discrepancy between technological capability and real-world implementation. Current regulatory frameworks often lag behind scientific innovation, failing to fully exploit rapid diagnostics or to adequately address critical gaps, such as the VBNC blind spot. Furthermore, the absence of global standardization, coupled with economic barriers that prevent low- and middle-income countries from adopting high-tech surveillance, results in a fragmented global defense system against a borderless AMR threat.

Ultimately, mitigating AMR requires far more than isolated interventions but it demands a unified One Health approach that effectively bridges veterinary, environmental, and human health sectors. A comparative analysis of current detection frameworks indicates that while genotypic technologies offer unprecedented resolution, their universal adoption remains economically unfeasible for many regions. Therefore, the most effective trend identified in this synthesis points toward hybrid surveillance models: utilizing rapid, cost-effective biosensors for high-throughput, first-line screening at processing facilities, while reserving WGS for complex epidemiological investigations. Future efforts must prioritize the harmonization of molecular detection protocols, the development of cost-effective and deployable rapid sensing technologies, and the enforcement of strict antimicrobial stewardship and biofilm control programs from farm to fork. Only by closing the loop between detection, data, integration, and timely regulatory action can the integrity of the global food supply be safeguarded against the rising challenge of antimicrobial resistance.

## Figures and Tables

**Table 1:** Conventional methods used for AMR detection.

Method	Principle	Type of Result	Key Features	References
Broth Microdilution (BMD)	Bacteria are inoculated into wells with Mueller-Hinton broth containing varying antibiotic concentrations.	Quantitative (MIC value)	Gold Standard (ISO 20776-1/CLSI M07). Automated and highly standardized. Essential for harmonized surveillance data.	[9,63,74,75,76]
Disk Diffusion (Kirby-Bauer)	Antibiotic disks are placed on an inoculated agar plate; zone of inhibition is measured after incubation.	Qualitative (Susceptible/Resistant)	Low cost and simple execution. Widely used in clinical/smaller labs. Lacks precise MIC values for risk assessment.	[77,78,79]
Gradient Strip Method (E-test)	Use of plastic strips with a pre-defined concentration gradient of the antibiotic.	Quantitative (MIC value)	Combines diffusion simplicity with quantitative MIC. Used to confirm ambiguous results. Ideal for fastidious organisms (e.g., *Campylobacter* spp.).	[76,80]

**Table 2:** Comparison of the available methods used for AMR detection in the food chain.

Feature	Conventional Phenotypic Methods	PCR Methods	WGS	Metagenomics	Rapid Technologies (Biosensors/MALDI)
Primary Target	Phenotypic expression (growth/inhibition)	Specific DNA sequences (ARGs)	Entire genome (Genotype & Phylogeny)	Total community DNA (resistome)	Proteins/Electrochemical signals
Required Time	Slow (2–4 days)	Rapid (hours)	Moderate (days)	Moderate (days to weeks)	Very Rapid (minutes to hours)
Throughput	High (with automation)	High (multiplexing)	High (batch sequencing)	High	Variable (often single sample)
Detects Unknown Resistance?	Yes (if phenotype is expressed)	No (requires known primers)	Yes (novel mutations/genes)	Yes	Generally, No (targeted)
Viability Discrimination	Excellent (only grows live cells)	Poor (detects dead cells unless v-PCR used)	Poor (DNA based)	Poor (DNA based)	Variable (depends on method)
Matrix Interference	Low (purification via culture)	High (inhibitors in food)	High (host DNA contamination)	Very High (host DNA dominance)	Moderate
Cost	Low	Moderate	High (but decreasing)	Very High	Variable (High setup, low per-test)
Regulatory Status	Gold Standard (ISO 20776)	Screening/Research	Emerging Standard (FDA/EURL)	Research/Exploratory	Screening/Confirmation
Key Limitation	Misses VBNC; slow to get results	False positives from dead cells; limited targets	Bioinformatics bottleneck; cost; genotype-phenotype mismatch	Host DNA contamination; biological noise; data complexity	Lack of comprehensive databases; regulatory validation

**Table 3:** Key alternative approaches to AMR prevention.

Strategy	Mechanism of Action	Key Impacts & Evidence	References
Vaccination Programs	Induces specific immunity and prevents disease transmission and outbreaks.	High efficacy, being considered a top-tier alternative. A major outcome is the improved performance metrics and reduced need for therapeutic treatments.	[158,159,160]
Probiotics and Prebiotics	Promotes beneficial gut flora and causes a competitive exclusion of harmful microbes.	Limit gastrointestinal infections, increasing gut health, and lowers overall demand for antimicrobial interventions.	[152,161,162]
Organic Acids and Acidulants	Acidifies gut environment and creates hostile conditions for pH-sensitive pathogens.	Reduce pathogenic load (such as *E. coli*) in the GI tract.	[163,164]
Antimicrobial Peptides (AMPs)	Targeted antimicrobial activity, while preserving normal microbiota (high specificity).	Cause a significant inhibition of pathogens like *Salmonella*, reducing diarrhea incidence and promotes growth comparable to antibiotics.	[165,166,167,168]
Plant-Derived Compounds & Essential Oils	Antimicrobial, antioxidant, anti-inflammatory and anti-quorum sensing (disrupts bacterial signaling).	Inhibit biofilm formation, reduce bacterial pathogenicity and virulence and causes no selection pressure for resistant strains.	[152,169,170]

## Data Availability

No new data were created or analyzed in this study. Data sharing is not applicable to this article.

## Supplementary Materials

The following supporting information can be downloaded at: https://www.mdpi.com/article/10.3390/antibiotics15030262/s1, Table S1. Standardized data extraction form utilized during the literature screening and data extraction process.

## Abbreviations

The following abbreviations are used in this manuscript:

AMR	Antimicrobial resistance
ARGs	Antimicrobial resistance genes
AST	Antimicrobial susceptibility testing
BMD	Broth microdilution
BPW	Buffered peptone water
CCPs	Critical control points
CLSI	Clinical and Laboratory Standards Institute
CRE	Carbapenem-resistant Enterobacteriaceae
CRISPR	Clustered regularly interspaced short palindromic repeats
dPCR	Digital polymerase chain reaction
ECOFFs	Epidemiological cut-off values
EFSA	European Food Safety Authority
EPS	Extracellular polymeric substances
ESBL	Extended-spectrum β-lactamase
EUCAST	European Committee on Antimicrobial Susceptibility Testing
EURL-AR	European Union Reference Laboratory for Antimicrobial Resistance
HGT	Horizontal gene transfer
HICs	High-income countries
LMICs	Low- and middle-income countries
MALDI-TOF MS	Matrix-assisted laser desorption/ionization time-of-flight mass spectrometry
MIC	Minimum inhibitory concentration
MRSA	Methicillin-resistant *Staphylococcus aureus*
NGS	Next-generation sequencing
PCR	Polymerase chain reaction
POC	Point-of-care
PMA	Propidium monoazide
QS	Quorum sensing
qPCR	Quantitative polymerase chain reaction
SNPs	Single-nucleotide polymorphisms
VBNC	Viable but non-culturable
VRE	Vancomycin-resistant enterococci
WASH	Water, sanitation and hygiene
WGS	Whole genome sequencing
WHO	World Health Organization
WWTPs	Wastewater treatment plants
